# The Optical Forces and Torques Exerted by Airy Light-Sheet on Magnetic Particles Utilized for Targeted Drug Delivery

**DOI:** 10.3390/mi15111369

**Published:** 2024-11-12

**Authors:** Ningning Song, Shiguo Chen, Hao Wang, Xinbo He, Bing Wei, Renxian Li, Shu Zhang, Lei Xu

**Affiliations:** 1 School of Physics, Xidian University, Xi’an 710071, China; nnsong@stu.xidian.edu.cn (N.S.); hexinbo@xidian.edu.cn (X.H.); rxli@mail.xidian.edu.cn (R.L.); zhangs@stu.xidian.edu.cn (S.Z.); 2 Key Laboratory of Optoelectronic Information Perception in Complex Environment, Ministry of Education, Xidian University, Xi’an 710071, China; 3 Xi’an Institute of Electromechanical Information Technology, Xi’an 710065, China

**Keywords:** airy light-sheet, magnetic particles, magnetic targeted drug delivery, optical radiation force, ssOptical spin torque

## Abstract

The remarkable properties of magnetic nanostructures have sparked considerable interest within the biomedical domain, owing to their potential for diverse applications. In targeted drug delivery systems, therapeutic molecules can be loaded onto magnetic nanocarriers and precisely guided and released within the body with the assistance of an externally applied magnetic field. However, conventional external magnetic fields generated by permanent magnets or electromagnets are limited by finite magnetic field gradients, shallow penetration depths, and low precision. The novel structured light field known as the Airy light-sheet possesses unique characteristics such as non-diffraction, self-healing, and self-acceleration, which can potentially overcome the limitations of traditional magnetic fields to some extent. While existing studies have primarily focused on the manipulation of dielectric particles by Airy light-sheet, comprehensive analyses exploring the intricate interplay between Airy light-sheet and magnetic nanostructures are currently lacking in the literature, with only preliminary theoretical discussions available. This study systematically explores the mechanical response of magnetic spherical particles under the influence of Airy light-sheet, including radiation forces and spin torques. Furthermore, we provide an in-depth analysis of the effects of particle size, permittivity, permeability, and incident light-sheet parameters on the mechanical effects. Our research findings not only offer new theoretical guidance and practical references for the application of magnetic nanoparticles in biomedicine but also provide valuable insights for the manipulation of other types of micro/nanoparticles using structured light fields.

## 1. Introduction

The flourishing development of nanotechnology has injected new vitality into the field of biomedicine, with the application prospects of magnetic nanoparticles attracting particular attention [1]. Owing to their unique physical properties and biocompatibility, magnetic nanoparticles exhibit broad application potential in targeted drug/gene delivery [2,3], magnetic resonance imaging (MRI) enhancement [4,5], and cancer treatment [6]. In targeted drug delivery systems, therapeutic molecules can be loaded onto magnetic nanocarriers and precisely guided and released within the body with the assistance of an external magnetic field, paving a new path for efficient disease treatment with minimal side effects [2].

Researchers have been exploring the use of external magnetic fields to guide and concentrate magnetic nanoparticle-based drug carriers to target sites like tumors. Traditional permanent magnets or electromagnets face limitations like limited magnetic field gradients, shallow penetration depths, and low precision [7,8,9,10,11,12]. To overcome this, novel approaches using superconducting magnets [13,14] and time-varying, spatially varying magnetic fields are being investigated [15]. Superconducting magnets can generate much higher field gradients, enabling the accumulation of magnetic nanocarriers even against high blood flow velocities [14]. Spatially varying fields improve drug targeting by enhancing nanoparticle accumulation at the target site. Additionally, time-varying magnetic fields are utilized to trigger drug release from thermally sensitive nanocarriers [15]. Challenges remain in optimizing magnetic properties, improving spatial specificity, and cost-effective system scale-up. Overall, advanced magnetic field systems hold promise for enhancing the efficacy and precision of magnetic drug targeting [12,14,15,16,17].

To overcome the bottleneck of traditional magnetic field control techniques, it is necessary to explore new methods for manipulating micro-/nanoparticles using structured light fields. Exhibiting distinctive characteristics, the Airy light-sheet represents an innovative structured illumination modality. This unique optical field possesses remarkable properties, such as its ability to resist diffraction, self-reconstruct upon encountering obstacles, and undergo self-acceleration during propagation [18,19,20,21]. During propagation, its transverse intensity distribution remains unchanged, while its trajectory exhibits a unique curved path along the propagation direction, displaying propagation behavior starkly different from traditional Gaussian beams. This unique light field characteristic endows the Airy light-sheet with tremendous potential in micro/nanoparticle manipulation, enabling the confinement and transportation of particles along specific paths without the need for external forces, thus opening up new possibilities for precise nanoparticle control.

Therefore, investigating the micro-manipulation of magnetic particles by Airy light sheet structured optical fields is of paramount importance. Relevant studies in the field of structured light beams have laid the theoretical foundation for this work. For example, Forbes et al. provided a comprehensive review of structured light [22], while Otte et al. summarized the applications of structured optical fields in optical control [23]. Forbes et al. discussed the fundamental principles of structured light, its interdisciplinary applications, recent advancements, and future challenges; Otte and Denz, on the other hand, focused on applications of structured light in optical trapping and manipulation, especially in the control of three-dimensional and four-dimensional structured light. These two reviews highlight the crucial role and developmental potential of structured light in modern optics. In terms of Airy beam research, Siviloglou et al. experimentally verified the acceleration characteristics of Airy beams, observing the non-diffractive and self-accelerating propagation of Airy beams in free space, thus confirming theoretical predictions [24,25]. Baumgartl demonstrated the application of Airy beams in optical micromanipulation, particularly in removing particles and cells from sample chambers [26]. Kim achieved lateral spin control of submicron spheres using two counter-propagating Airy beams [27]; Lu et al. further derived the theoretical optical forces exerted by one-dimensional Airy beams on dielectric cylindrical particles [28]. Additionally, Li, Mitri, and other researchers have explored the mechanical properties of magnetic particles under Bessel beams [29,30,31]. In reference [29], Mitri analyzed the influence of beam polarization and topological charge on the optical pulling force exerted on Rayleigh spheres; in reference [30], they discussed the optical pulling force and torque on semiconductor ellipsoids under Bessel beams. Li et al. investigated the optical torque, including spin and orbital torque, on magnetically absorbing spheres under varying polarization conditions of Bessel beams. Furthermore, Barton et al. proposed a theory for the net radiation force and torque exerted by arbitrary light fields on spherical particles [32]. Mitri also preliminarily explored the feasibility of using Airy beams to manipulate magnetic nanoparticles, deriving the longitudinal and transverse photo-induced asymmetry factors (PAFs) for magnetic cylinders in arbitrary wavefronts and polarized light fields, and discussed their applications in optical forces and torque [33].

However, existing research has primarily focused on the manipulation of dielectric particles by Airy beams, while studies on magnetic particles have been limited to simple theoretical explorations. A systematic investigation of the interaction between Airy light-sheet and magnetic spherical particles remains to be conducted. Therefore, building upon previous work, this study aims to delve into the interaction mechanism between Airy light-sheet and magnetic spherical particles and unveil their potential applications in magnetic targeted drug delivery systems. This endeavor is expected to provide new theoretical guidance and practical references for technological development in this field.

Based on the Maxwell stress tensor theory and the generalized Lorenz–Mie theory, this study systematically investigates the mechanical responses, including radiation forces and spin torques, experienced by magnetic spherical particles under the influence of Airy light-sheet. Furthermore, it provides an in-depth analysis of the impact of particle size, permittivity, permeability, and incident light field parameters on these mechanical effects. Our research findings not only extend new perspectives for the application of magnetic nanoparticles in the biomedical field but also provide valuable references for the manipulation of other types of micro/nanoparticle systems using structured light fields.

## 2. Methods

**Figure 1:**
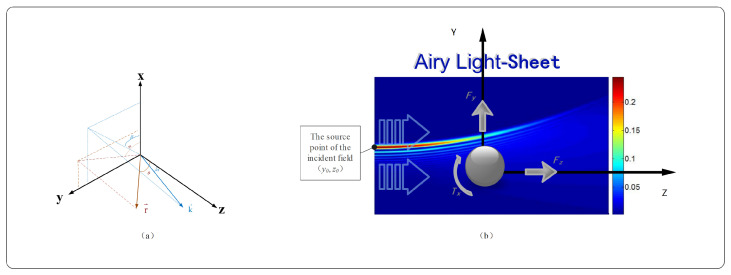
Schematic illustration of Airy light-sheet propagation in space. (**a**) The definition of the wave vector $\vec{k}$, the coordinate vector $\vec{r}$, and its angle with the coordinate axis. (**b**) Schematic representation of the force and torque acting on magnetic spherical particles in an Airy light field.

As illustrated in Figure 1b, a magnetic spherical particle is subjected to force and torque when positioned in an Airy light-sheet field. With the particle center as the origin of a Cartesian coordinate system, the source point of the incident Airy light-sheet field is located at $\left(y_{0},z_{0}\right)$. The particle experiences a scattering force $F_{z}$ along the propagation direction of the light, a gradient force $F_{y}$ perpendicular to the propagation direction, and an optical spin torque (OST) $T_{x}$ that causes the particle to rotate about its own axis. Orbital angular momentum only exists in three-dimensional beams. Therefore, the Airy light-sheet does not possess orbital angular momentum, and consequently, there is no optical orbital torque. Furthermore, the optical spin torque (OST) is only present in the case of a TM-polarized incident Airy light-sheet, where the optical spin torque components $T_{y}$ and $T_{z}$ are zero, while $T_{x}$ is non-zero. In this study, we investigate the distinct polarization configurations of the Airy light-sheet, specifically examining the transverse electric (TE) and transverse magnetic (TM) cases. In the transverse electric (TE) polarization scenario, the non-vanishing field components are $E_{x}$, $H_{y}$, and $H_{z}$. Specifically, the electric field component $E_{x}$ is polarized along the *x*-axis, while the magnetic field components $H_{y}$ and $H_{z}$ are oriented along the *y*- and *z*-axes, respectively. Conversely, for the transverse magnetic (TM) polarization case, the existing field components are $H_{x}$, $E_{y}$, and $E_{z}$. Here, the magnetic field component $H_{x}$ is polarized along the *x*-axis, while the electric field components $E_{y}$ and $E_{z}$ are aligned with the *y*- and *z*-axes, respectively. In the theoretical derivations and numerical simulations presented in this paper, we assume that the environment surrounding the particle is a lossless, isotropic, and homogeneous vacuum.

### 2.1. Optical Force

When light interacts with particles, momentum transfer occurs. According to the principle of momentum conservation, the particle experiences an optical radiation force (OF) and an optical radiation torque (OT). The rates of change in linear and angular momenta correspond to OF and OT, respectively [34,35]. By integrating the time-averaged Maxwell stress tensor over a virtual surface enclosing the scattering body, we can precisely calculate the radiation force exerted on a spherical particle as follows:

(1) $$F=\oint_{S}\hat{n}\cdot \langle \overset{↔}{\;K}\rangle dS$$

The vector quantity represented by the symbol $\hat{n}$ denotes the outward-pointing unit normal vector defined on the surface enclosing the spherical structure under consideration. The symbol *S* represents an arbitrary closed surface enclosing the particle under investigation. The quantity $\langle \overset{↔}{\;K}\rangle$ denotes the time-averaged Maxwell stress tensor, defined as

(2) K↔=12ReεEE*+μHH*−12εE·E*+μH·H*I↔

Re denotes taking the real part and Im represents taking the imaginary part. The symbols ε and μ denote the permittivity and permeability, respectively, of the medium surrounding the particle under investigation. The superscript asterisk (*) represents the complex conjugate operation. The tensor quantity I is the identity tensor, a mathematical construct that preserves the directional properties of vector fields. The vector fields E and H represent the total electric and magnetic fields, respectively, present in the surrounding medium, encompassing both the incident and scattered field contributions. In the specific scenario where the background medium is lossless, the Cartesian components of the optical force exerted on the particle can be expressed as follows:

(3) Fx=ReF1,Fy=ImF1,Fz=ReF2

where

(4) $$\begin{array}{c} F_{1}=\frac{2\pi \varepsilon }{k^{2}}|E_{0}{|}^{2}\sum_{n=1}^{\infty }\sum_{m=-n}^{n}\left\{\frac{{\left[\left(n-m)(n+m+1\right)\right]}^{\frac{1}{2}}}{n(n+1)}\times \left(\begin{array}{c} {\tilde{a}}_{m,n}^{pol}{\tilde{b}}_{m+1,n}^{*pol}+{\tilde{b}}_{m,n}^{pol}{\tilde{a}}_{m+1,n}^{*pol}\\ -{\tilde{p}}_{m,n}^{pol}{\tilde{q}}_{m+1,n}^{*pol}-{\tilde{q}}_{m,n}^{pol}{\tilde{p}}_{m+1,n}^{*pol} \end{array}\right)\right.\\ -{\left[\frac{n(n+2)(n+m+1)(n+m+2)}{{\left(n+1\right)}^{2}\left(2n+1\right)\left(2n+3\right)}\right]}^{\frac{1}{2}}\times \left(\begin{array}{c} {\tilde{a}}_{m,n}^{pol}{\tilde{a}}_{m+1,n+1}^{*pol}+{\tilde{b}}_{m,n}^{pol}{\tilde{b}}_{m+1,n+1}^{*pol}\\ -{\tilde{p}}_{m,n}^{pol}{\tilde{p}}_{m+1,n+1}^{*pol}-{\tilde{q}}_{m,n}^{pol}{\tilde{q}}_{m+1,n+1}^{*pol} \end{array}\right)\\ \left.+{\left[\frac{n(n+2)(n-m)(n-m+1)}{{\left(n+1\right)}^{2}\left(2n+1\right)\left(2n+3\right)}\right]}^{\frac{1}{2}}\times \left(\begin{array}{c} {\tilde{a}}_{m,n+1}^{pol}{\tilde{a}}_{m+1,n}^{*pol}+{\tilde{b}}_{m,n+1}^{pol}{\tilde{b}}_{m+1,n}^{*pol}\\ -{\tilde{p}}_{m,n+1}^{pol}{\tilde{p}}_{m+1,n}^{*pol}-{\tilde{q}}_{m,n+1}^{pol}{\tilde{q}}_{m+1,n}^{*pol} \end{array}\right)\right\} \end{array}$$

(5) $$\begin{array}{c} F_{2}=-\frac{4\pi \varepsilon }{k^{2}}|E_{0}{|}^{2}\sum_{n=1}^{\infty }\sum_{m=-n}^{n}\left\{\frac{m}{n(n+1)}\times \left({\tilde{a}}_{m,n}^{pol}{\tilde{b}}_{m,n}^{*pol}-{\tilde{p}}_{m,n}^{pol}{\tilde{q}}_{m,n}^{*pol}\right)\right.\;\;\;\;\;\;\;\;\;\;\;\\ \left.+{\left[\frac{n(n+2)(n-m+1)(n+m+1)}{{\left(n+1\right)}^{2}\left(2n+1\right)\left(2n+3\right)}\right]}^{\frac{1}{2}}\times \left(\begin{array}{c} {\tilde{a}}_{m,n}^{pol}{\tilde{a}}_{m,n+1}^{*pol}+{\tilde{b}}_{m,n}^{pol}{\tilde{b}}_{m,n+1}^{*pol}\\ -{\tilde{p}}_{m,n}^{pol}{\tilde{p}}_{m,n+1}^{*pol}-{\tilde{q}}_{m,n}^{pol}{\tilde{q}}_{m,n+1}^{*pol} \end{array}\right)\right\} \end{array}$$

and

(6) $$\begin{array}{c} {\tilde{a}}_{m,n}^{pol}=a_{m,n}^{pol}-\frac{1}{2}p_{m,n}^{pol},\;\;\;\;\;\;\;\;\;\;\;\;\;{\tilde{p}}_{m,n}^{pol}=\frac{1}{2}p_{m,n}^{pol}\\ {\tilde{b}}_{m,n}^{pol}=b_{m,n}^{pol}-\frac{1}{2}q_{m,n}^{pol},\;\;\;\;\;\;\;\;\;\;\;\;\;{\tilde{q}}_{m,n}^{pol}=\frac{1}{2}q_{m,n}^{pol} \end{array}$$

(7) $$\begin{array}{c} a_{m,n}^{pol}=a_{n}p_{m,n}^{pol},\;\;\;\;\;\;\;\;\;\;\;\;\;\;\;\;\;\;\;b_{m,n}^{pol}=b_{n}q_{m,n}^{pol}\\ c_{m,n}^{pol}=c_{n}q_{m,n}^{pol},\;\;\;\;\;\;\;\;\;\;\;\;\;\;\;\;\;\;\;d_{m,n}^{pol}=d_{n}p_{m,n}^{pol} \end{array}$$

where k=2π/λ, the symbol λ represents the wavelength of the incident electromagnetic field illuminating the system. The quantity $E_{0}$ denotes the amplitude of the incident electric field vector. The beam shape coefficients (BSCs) of the incident Airy light-sheet are represented by the symbols $p_{m,n}^{pol}$ and $q_{m,n}^{pol}$, where the superscript “pol” indicates the specific polarization state under consideration, which are given as follows:

(8) $$\begin{array}{c} p_{m,n}^{TE}=i\frac{k}{E_{0}}\sqrt{D_{mn}}\left\{\begin{array}{c} e^{-i\frac{\pi }{2}\cos\alpha }\int_{0}^{\frac{\pi }{2}}\pi_{mn}\left(\cos\alpha \right)A_{x}^{E}\left(\alpha \right)e^{-ik\cos\alpha z_{0}}\cos\alpha d\alpha \\ -e^{i\frac{\pi }{2}\cos\alpha }\int_{-\frac{\pi }{2}}^{0}\pi_{mn}\left(\cos\alpha \right)A_{x}^{E}\left(\alpha \right)e^{-ik\cos\alpha z_{0}}\cos\alpha d\alpha \end{array}\right\} \end{array}$$

(9) $$\begin{array}{c} q_{m,n}^{TE}=i\frac{k}{E_{0}}\sqrt{D_{mn}}\left\{\begin{array}{c} e^{-i\frac{\pi }{2}\cos\alpha }\int_{0}^{\frac{\pi }{2}}\tau_{mn}\left(\cos\alpha \right)A_{x}^{E}\left(\alpha \right)e^{-ik\cos\alpha z_{0}}\cos\alpha d\alpha \\ -e^{i\frac{\pi }{2}\cos\alpha }\int_{-\frac{\pi }{2}}^{0}\tau_{mn}\left(\cos\alpha \right)A_{x}^{E}\left(\alpha \right)e^{-ik\cos\alpha z_{0}}\cos\alpha d\alpha \end{array}\right\} \end{array}$$

(10) $$\begin{array}{c} p_{m,n}^{TM}=-\frac{k}{H_{0}}\sqrt{D_{mn}}\left\{\begin{array}{c} e^{-i\frac{\pi }{2}\cos\alpha }\int_{0}^{\frac{\pi }{2}}\tau_{mn}\left(\cos\alpha \right)A_{x}^{H}\left(\alpha \right)e^{-ik\cos\alpha z_{0}}\cos\alpha d\alpha \\ -e^{i\frac{\pi }{2}\cos\alpha }\int_{-\frac{\pi }{2}}^{0}\tau_{mn}\left(\cos\alpha \right)A_{x}^{H}\left(\alpha \right)e^{-ik\cos\alpha z_{0}}\cos\alpha d\alpha \end{array}\right\} \end{array}$$

(11) $$\begin{array}{c} q_{m,n}^{TM}=-\frac{k}{H_{0}}\sqrt{D_{mn}}\left\{\begin{array}{c} e^{-i\frac{\pi }{2}\cos\alpha }\int_{0}^{\frac{\pi }{2}}\pi_{mn}\left(\cos\alpha \right)A_{x}^{H}\left(\alpha \right)e^{-ik\cos\alpha z_{0}}\cos\alpha d\alpha \\ -e^{i\frac{\pi }{2}\cos\alpha }\int_{-\frac{\pi }{2}}^{0}\pi_{mn}\left(\cos\alpha \right)A_{x}^{H}\left(\alpha \right)e^{-ik\cos\alpha z_{0}}\cos\alpha d\alpha \end{array}\right\} \end{array}$$

where

(12) $$D_{mn}=\frac{(2n+1)(n-m)!}{n(n+1)(n+m)!}$$

(13) $$\pi_{mn}\left(\cos\alpha \right)=m\frac{P_{n}^{m}\left(\cos\alpha \right)}{\sin\alpha },\tau_{mn}\left(\cos\alpha \right)=\frac{dP_{n}^{m}\left(\cos\alpha \right)}{d\alpha }$$

$P_{n}^{m}\left(\cos\alpha \right)$ is the associated Legendre function, and the quantities $A_{x}^{E}\left(\alpha \right)$ and $A_{x}^{H}\left(\alpha \right)$ represent the angular spectrum distributions associated with the transverse electric (TE) and transverse magnetic (TM) polarization configurations of the Airy light-sheet, respectively.

(14) $$\begin{array}{c} A_{x}^{E}\left(\cos\alpha \right)=E_{0}\frac{w_{0}}{2\pi }\exp\left[\frac{{\left(\gamma -ikw_{0}\cos\alpha \right)}^{3}}{3}\right]e^{-ik_{y}y_{0}}\;\\ A_{x}^{H}\left(\cos\alpha \right)=H_{0}\frac{w_{0}}{2\pi }\exp\left[\frac{{\left(\gamma -ikw_{0}\cos\alpha \right)}^{3}}{3}\right]e^{-ik_{y}y_{0}} \end{array}$$

The parameters $w_{0}$ and γ govern the spatial distribution and propagation characteristics of the Airy light-sheet, respectively. Specifically, $w_{0}$ represents the characteristic transverse scale that dictates the lateral confinement and spatial extent of the beam. Conversely, γ is the attenuation parameter, which influences the rate at which the beam’s intensity decays along the propagation axis. In Equation (7), $a_{n}$, $b_{n}$, $c_{n}$, and $d_{n}$ are the classical Mie scattering coefficients [36,37].

(15) $$\begin{array}{c} a_{n}=\frac{\mu_{sp}\Psi_{n}\left(x\right)\Psi_{n}^{\prime }\left(Mx\right)-\mu M\Psi_{n}^{\prime }\left(x\right)\Psi_{n}\left(Mx\right)}{\mu_{sp}\xi_{n}\left(x\right)\Psi_{n}^{\prime }\left(Mx\right)-\mu M\xi_{n}^{\prime }\left(x\right)\Psi_{n}\left(Mx\right)}\;\\ b_{n}=\frac{\mu M\Psi_{n}\left(x\right)\Psi_{n}^{\prime }\left(Mx\right)-\mu_{sp}\Psi_{n}^{\prime }\left(x\right)\Psi_{n}\left(Mx\right)}{\mu M\xi_{n}\left(x\right)\Psi_{n}^{\prime }\left(Mx\right)-\mu_{sp}\xi_{n}^{\prime }\left(x\right)\Psi_{n}\left(Mx\right)}\;\\ c_{n}=\frac{M\mu_{sp}\left[\xi_{n}\left(x\right)\Psi_{n}^{\prime }\left(x\right)-\xi_{n}^{\prime }\left(x\right)\Psi_{n}\left(x\right)\right]}{\mu_{sp}\xi_{n}\left(x\right)\Psi_{n}^{\prime }\left(Mx\right)-\mu M\xi_{n}^{\prime }\left(x\right)\Psi_{n}\left(Mx\right)}\;\\ d_{n}=\frac{\mu M^{2}\left[\xi_{n}\left(x\right)\Psi_{n}^{\prime }\left(x\right)-\xi_{n}^{\prime }\left(x\right)\Psi_{n}\left(x\right)\right]}{\mu M\xi_{n}\left(x\right)\Psi_{n}^{\prime }\left(Mx\right)-\mu_{sp}\xi_{n}^{\prime }\left(x\right)\Psi_{n}\left(Mx\right)} \end{array}$$

where $\mu_{sp}$ and μ are the magnetic permeabilities of the particle and the surrounding medium, respectively. *M* is the refractive index of the particle. $\Psi_{n}\left(\cdot \right)$ and $\xi_{n}\left(\cdot \right)$ are the Riccati–Bessel functions. x=ka is the particle’s size parameter, with *k* being the wave number and *a* being the particle’s radius.

### 2.2. Optical Torque

Akin to the formulation of optical radiation forces, the expression for optical radiation torques can be derived by employing the Maxwell stress tensor approach. Grounded in the principle of angular momentum conservation, the radiation torque exerted on a particle of arbitrary geometry is equivalent to the time-averaged rate of change in angular momentum imparted by the incident electromagnetic field [38,39].

(16) $$T=-\oint_{S}\hat{n}\cdot \left(\langle \overset{↔}{\;K}\rangle \times \vec{r}\right)dS$$

The OST components are

(17) Tx=ReN1,Ty=ImN1,Tz=ReN2

where

(18) $$\begin{array}{c} N_{1}=\frac{2\pi \varepsilon_{0}}{k^{3}}{\left|E_{0}\right|}^{2}\sum_{n=1}^{\infty }\sum_{m=-n}^{n}\left\{\begin{array}{c} {\left[(n-m)(n+m+1)\right]}^{\frac{1}{2}}\\ \times \left({\tilde{a}}_{m,n}^{\mathrm{pol}}{\tilde{a}}_{m+1,n}^{*pol}+{\tilde{b}}_{m,n}^{\mathrm{pol}}{\tilde{b}}_{m+1,n}^{*\mathrm{pol}}-{\tilde{p}}_{m,n}^{\mathrm{pol}}{\tilde{p}}_{m+1,n}^{*pol}-{\tilde{q}}_{m,n}^{pol}{\tilde{q}}_{m+1,n}^{*pol}\right) \end{array}\right\}\\ N_{2}=-\frac{2\pi \varepsilon_{0}}{k^{3}}{\left|E_{0}\right|}^{2}\sum_{n=1}^{\infty }\sum_{m=-n}^{n}m\left(|{\tilde{a}}_{m,n}^{\mathrm{pol}}{|}^{2}+|{\tilde{b}}_{m,n}^{\mathrm{pol}}{|}^{2}-\left|{\tilde{p}}_{m,n}^{\mathrm{pol}}{|}^{2}-\right|{\tilde{q}}_{m,n}^{pol}{|}^{2}\right) \end{array}$$

## 3. Results

This section presents the numerical simulations conducted in this study, with a primary focus on the comparative analysis of optical radiation forces and torques exerted by an Airy light-sheet on dielectric and magneto-dielectric spherical particles. We systematically explore the impact of various factors, including the particle’s radius, its electromagnetic properties, the polarization state of the incident beam, as well as the transverse scale parameter $kw_{0}$ and the attenuation parameter γ, which govern the spatial distribution of the structured light field.

In optical tweezer systems, a near-infrared laser with a wavelength of 1.064 μm is typically chosen as the light source. At this wavelength, the absorption coefficient of biological samples is low, which can minimize photothermal damage to cells or biological tissues [2]. In magnetic targeted drug delivery systems, the most commonly used magnetic nanoparticles are ${\mathrm{Fe}}_{3}$$O_{4}$ (magnetite) nanoparticles. ${\mathrm{Fe}}_{3}$$O_{4}$ nanoparticles possess excellent magnetic responsiveness and biocompatibility, making them an ideal magnetic targeting carrier material. The electromagnetic property parameters of ${\mathrm{Fe}}_{3}$$O_{4}$ nanoparticles in an optical field with a wavelength of 1.064 μm are as follows: $\epsilon_{r}$ = 2.1112 + 0.3698i, $\mu_{r}$ = 1000. Therefore, unless otherwise specified, the following parameter settings are employed in the numerical simulations: the wavelength of the incident field is λ = 1.064 μm, the transverse scale parameter is $k\omega_{0}$ = 6.5, the attenuation parameter is γ = 0.1, and the electric field amplitude is $E_{0}$ = $1\times 10^{6}$ V/m. The object under study is a spherical particle with relative complex relative permittivity $\epsilon_{r}$ = 2.1112 + 0.3698i, relative permeability $\mu_{r}$ = 1000, and spatial coordinates $\left(y_{0},z_{0}\right)$ = (0, 0.25) μm.

**Figure 2:**
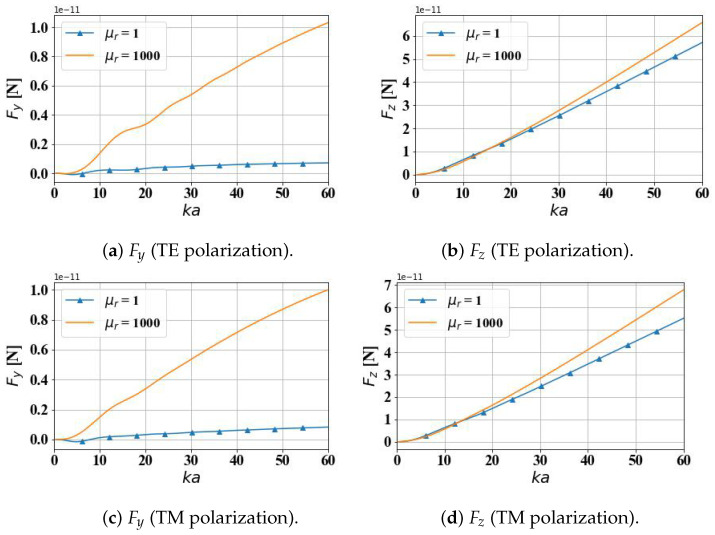
Comparison of optical force on dielectric ($\mu_{r}$ = 1) and magnetic ($\mu_{r}$ = 1000) spheres.

Figure 2 presents a comparative analysis of the optical radiation forces acting on a dielectric sphere ($\mu_{r}=1$) and a magnetic sphere ($\mu_{r}=1000$) when illuminated by an Airy light-sheet, with the forces measured in Newtons (N). Specifically, it examines the transverse gradient force $F_{y}$ and the longitudinal scattering force $F_{z}$ experienced by these spherical particles under structured illumination. This study explores the variation in these optical forces as the sphere radius increases, while considering the influence of the incident Airy light-sheet’s polarization state, whether transverse electric (TE) or transverse magnetic (TM). The calculation range for the optical size parameter ka spans from 0 to 60, corresponding to an actual spherical particle radius range of 0 to 10 μm. In other words, the maximum particle radius considered is approximately ten times the incident wavelength. As anticipated, the radiation forces exhibit an increasing trend with the particle radius ka, with the longitudinal scattering force $F_{z}$ displaying a more rapid growth rate. As the particle radius increases, the scattering cross-section of the particle (relative to the wavelength of light) also increases. This means the particle can intercept more photons, enhancing energy absorption and scattering. According to the Generalized Lorenz–Mie theory (GLMT), a particle’s scattering intensity and scattering force are related to its size. When the particle size approaches or exceeds the wavelength of light, the scattering cross-section increases significantly, enhancing the radiation force. The computed results reveal that the transverse gradient force $F_{y}$ experienced by the magnetic particle within the Airy light-sheet field is significantly larger than that of the dielectric particle, a highly advantageous characteristic for targeted drug delivery applications, highlighting the superiority of magnetic particles in this context. While the longitudinal scattering force $F_{z}$ acting on the magnetic particle is slightly larger than that on the dielectric particle, the difference is not substantial. Notably, the polarization state of the incident beam exerts minimal influence on the calculated results.

### 3.1. Optical Force

**Figure 3:**
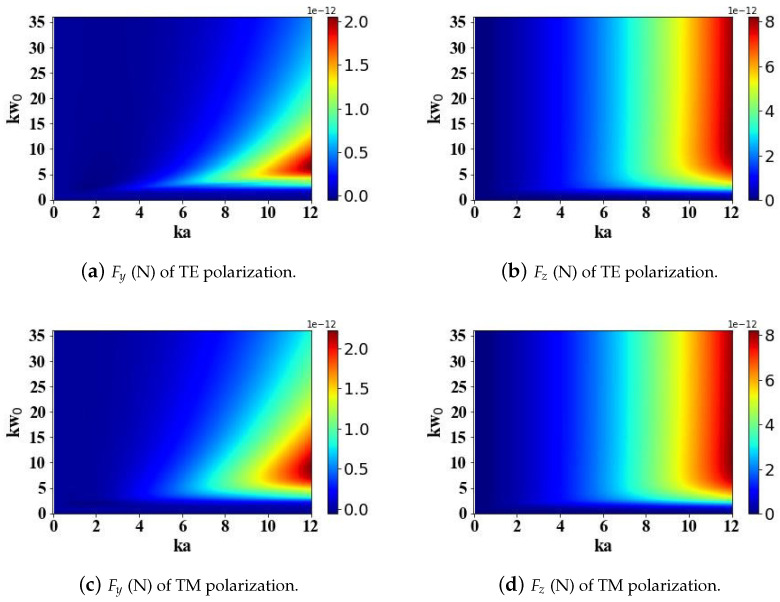
The influence of the transverse scale parameter $k\omega_{0}$ (ranging from 0 to 35) of the Airy light-sheet and the optical size parameter ka (ranging from 0 to 12) of the sphere on the optical radiation forces.

Figure 3 presents calculations of the optical radiation forces acting on a magneto-dielectric spherical particle when illuminated by an Airy light-sheet. Specifically, it quantifies the transverse gradient force $F_{y}$ and the longitudinal scattering force $F_{z}$ exerted on the particle by the structured electromagnetic field. The figure delves into the influence of two key parameters on the optical radiation forces experienced by the magneto-dielectric sphere: the beam transverse scale parameter $k\omega_{0}$, spanning a range from 0 to 35, and the optical size parameter ka, varying between 0 and 12. This study also examines the influence of the incident Airy light-sheet’s polarization state, considering both transverse electric (TE) and transverse magnetic (TM) configurations. According to the simulation outcomes, the transverse scale parameter $k\omega_{0}$ exerts a minor impact on the longitudinal scattering force $F_{z}$, while primarily influencing the transverse gradient force $F_{y}$. When the beam transverse scale parameter $k\omega_{0}$ is less than 3, the radiation forces are zero. As $k\omega_{0}$ continues to increase, the transverse gradient force $F_{y}$ first increases and then decreases, reaching a maximum when $k\omega_{0}$ is approximately 0.5 times ka. The simulation results reveal that the polarization configuration of the incident Airy light-sheet, whether transverse electric (TE) or transverse magnetic (TM), exerts a negligible influence on the computed optical radiation forces acting on the magneto-dielectric spherical particle.

**Figure 4:**
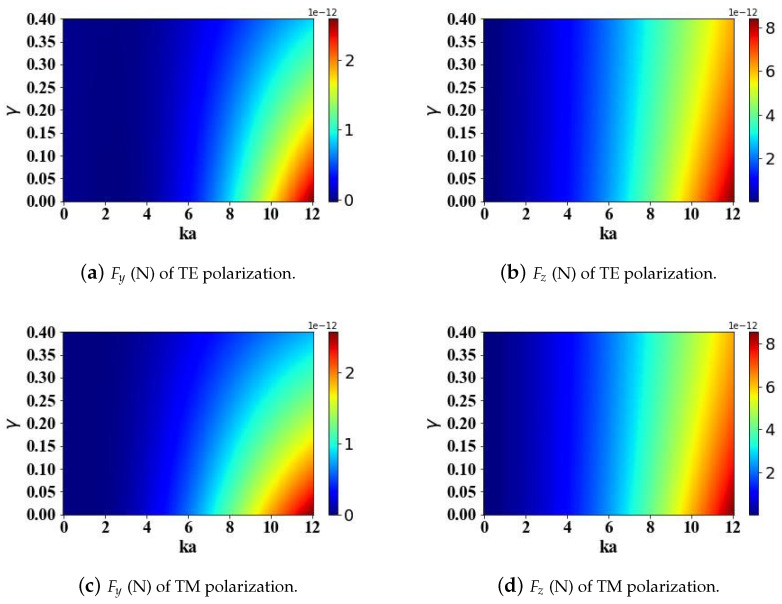
The influence of the beam attenuation parameter γ (ranging from 0 to 0.4) of the Airy light-sheet and the optical size parameter ka (ranging from 0 to 12) of the sphere on the optical radiation forces.

Figure 4 is similar to Figure 3, except that it analyzes the effects of attenuation parameter γ (ranging from 0 to 0.4) and optical size parameter ka (ranging from 0 to 12) on the results. The computational analysis reveals that an increase in the attenuation parameter is accompanied by a diminution in the amplitude of the electromagnetic field. Consequently, this attenuation manifests as a concomitant reduction in the optical radiation forces exerted on the particle. As the attenuation parameter decreases, the transverse gradient force $F_{y}$ decays faster than the longitudinal scattering force $F_{z}$. Therefore, the variation of γ has a greater impact on the transverse gradient force $F_{y}$ than on the longitudinal scattering force $F_{z}$. Similarly, akin to the observations regarding the attenuation parameter, the polarization configuration of the incident beam exerts a negligible impact on the computed optical radiation forces acting on the particle.

**Figure 5:**
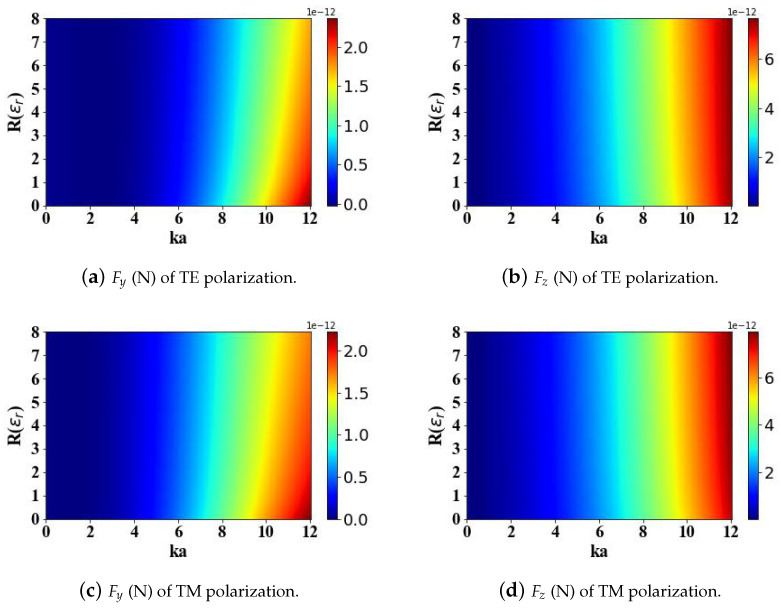
The influence of the real part of the relative permittivity of magnetic spherical particles (ranging from 0 to 8) and the optical size parameter ka (ranging from 0 to 12) on the optical radiation forces.

**Figure 6:**
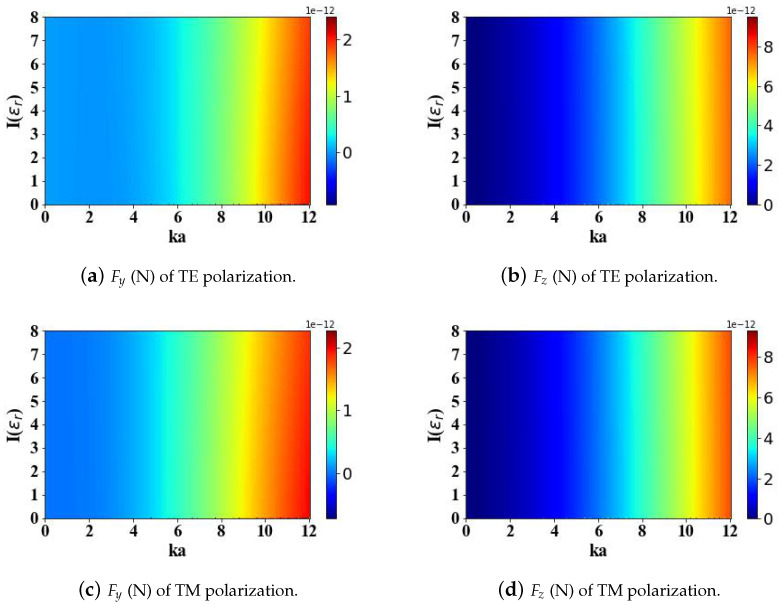
The influence of the imaginary part of the relative permittivity of magnetic spherical particles (ranging from 0 to 8) and the optical size parameter ka (ranging from 0 to 12) on the optical radiation forces.

The relative permittivity of magnetic spherical particles also influences the radiation force. Therefore, Figure 5 and Figure 6 discuss the effects of the real and imaginary parts (ranging from 0 to 8) of the relative permittivity of magnetic spherical particles on the radiation force when subjected to the structured electromagnetic field generated by an Airy light-sheet. An augmentation in the real component of the relative permittivity leads to a decrease in the transverse gradient force $F_{y}$, while the longitudinal scattering force $F_{z}$ increases, albeit both changes occur at a slow rate. Similarly, the computational analysis reveals that the polarization configuration of the incident illumination exerts a negligible influence on the calculated optical radiation forces acting on the particle.

The calculated results in Figure 6 show that as the optical size parameter ka of the spherical particle augments, the radiation forces $F_{y}$ and $F_{z}$ both gradually increase. This study reveals that variations in the imaginary component of the relative permittivity exert a negligible impact on the optical radiation forces experienced by the particle. Similarly, the polarization state of the incident beam has little impact on the calculated results.

**Figure 7:**
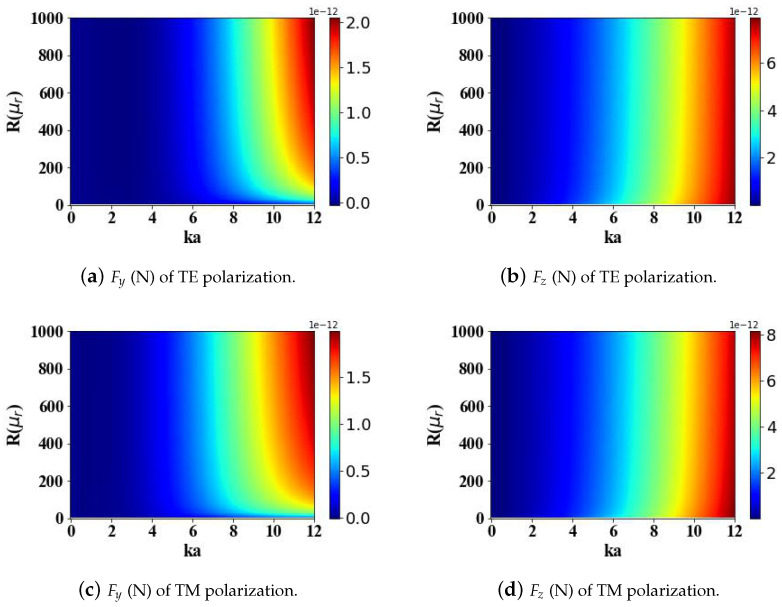
The influence of the real part of the relative permeability of magnetic spherical particles (ranging from 0 to 1000) and the optical size parameter ka (ranging from 0 to 12) on the optical radiation forces.

**Figure 8:**
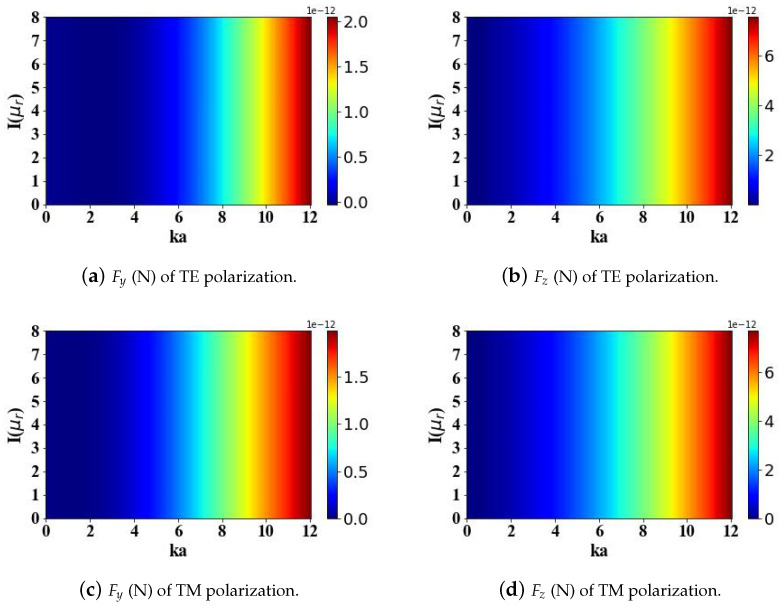
The influence of the imaginary part of the relative permeability of magnetic spherical particles (ranging from 0 to 8) and the optical size parameter ka (ranging from 0 to 12) on the optical radiation forces.

This study delves into the influence of the magnetic properties of the spherical particle on the optical radiation forces imparted by the structured electromagnetic field of an Airy light-sheet. Specifically, Figure 7 and Figure 8 explore the effects of the real and imaginary components, respectively, of the relative magnetic permeability on the resulting optical forces experienced by the particle. As the real part of the relative magnetic permeability increases, the transverse gradient force $F_{y}$ increases, while the longitudinal scattering force $F_{z}$ decreases slowly. The computational analysis reveals that when the real component of the relative magnetic permeability of the spherical particle is nullified, the transverse gradient force $F_{y}$ becomes negligible, approaching zero. Concurrently, the longitudinal scattering force $F_{z}$ maintains a non-zero value. This study reveals that the polarization state of the incident Airy light-sheet exerts a notable influence on the magnitudes of the optical radiation forces experienced by the particle. Specifically, when the Airy light sheet is transverse electric (TE) polarized, the maximum value of the transverse gradient force $F_{y}$ exhibits a larger magnitude compared to the case of a transverse magnetic (TM) polarized incident field. Conversely, the longitudinal scattering force $F_{z}$ attains a greater maximum value when the incident Airy light-sheet is TM-polarized, in contrast to the TE polarization configuration.

The results in Figure 8 show that the imaginary part of the relative magnetic permeability of the magnetic sphere ($\mu_{r}$ = 1000 + $m_{1}$i, where $m_{1}$ ranges from 0 to 8) does not affect the radiation forces experienced by the sphere. The computational analysis reveals that the transverse gradient force $F_{y}$ attains a greater maximum magnitude when the incident Airy light-sheet is transverse electric (TE) polarized, in comparison to the case of a transverse magnetic (TM) polarized incident field.

**Figure 9:**
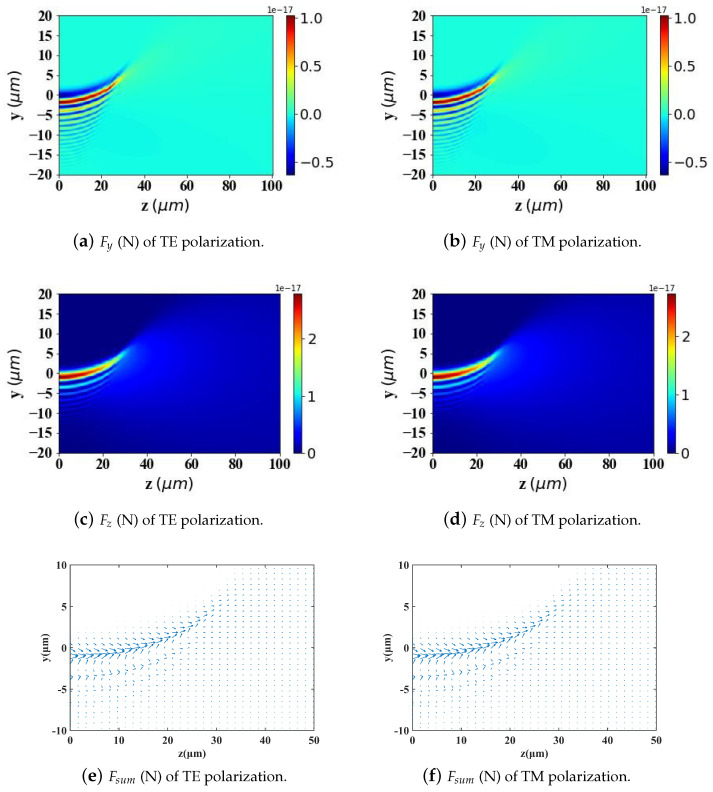
Two-dimensional spatial distribution of optical radiation forces exerted by an Airy light-sheet on a magnetic spherical particle (particle radius = 10 nm).

**Figure 10:**
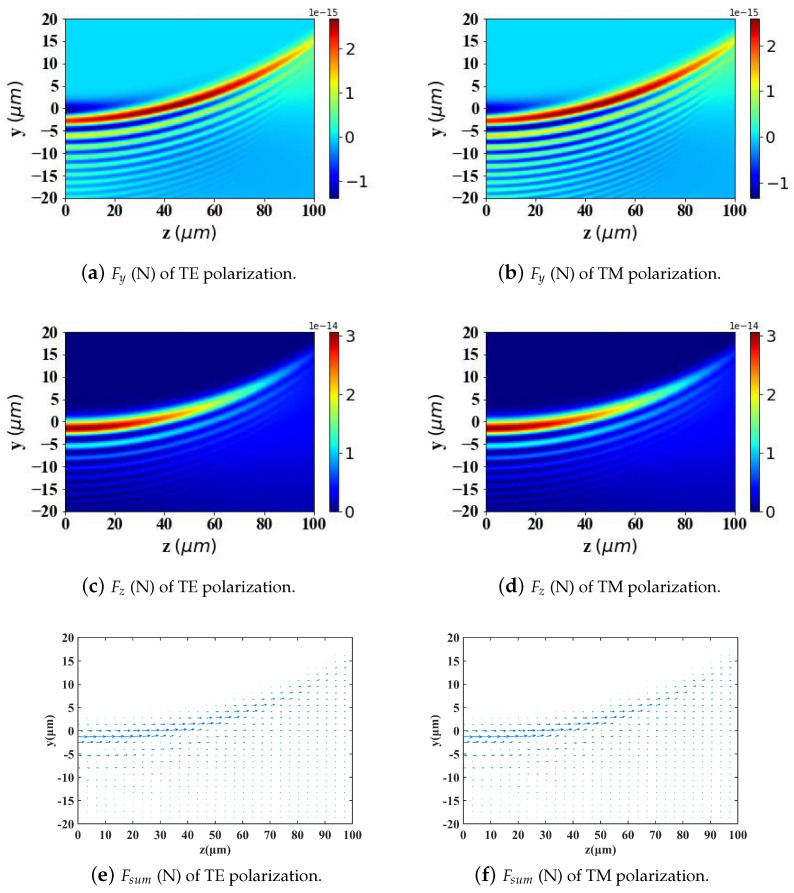
The same as in Figure 9 but magnetic spherical particle radius is 100 nm.

**Figure 11:**
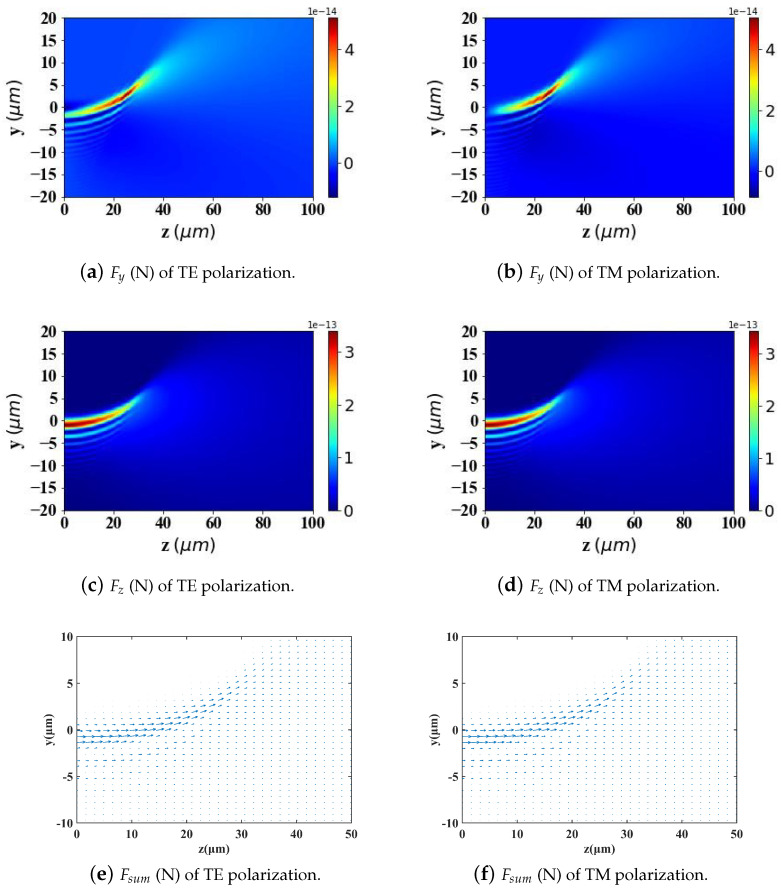
The same as in Figure 9 but magnetic spherical particle radius is 200 nm.

According to Ref. [2], to achieve better-targeting effects, the size of ${\mathrm{Fe}}_{3}$$O_{4}$ nanoparticles is typically controlled within the range of 10–200 nm. Nanoparticles within this size range can effectively penetrate tumor tissues while avoiding rapid clearance by macrophages. Particle sizes that are too large or too small will affect the efficiency of targeted delivery. Therefore, in Figure 9, Figure 10 and Figure 11, we analyze and study the spatial distribution of optical radiation forces in the yz-plane experienced by magnetic spherical particles with sizes of 10 nm, 100 nm, and 200 nm, respectively.

This study reveals that the optical radiation forces experienced by particles within the Airy light-sheet field exhibit a spatial distribution that is closely correlated with the energy concentration of the Airy structured beam. Specifically, the main lobe of the Airy light-sheet, where the electromagnetic energy is predominantly localized, exerts the most substantial optical forces on particles situated within this region. Similarly, larger spherical particles experience greater radiation forces. Interestingly, when the spherical particle size is too large (200 nm) or too small (10 nm), the radiation force values become very small in the region where *z* > 40 μm. However, for a spherical particle radius of 100 nm, there is a distribution of radiation force values in the same region. This pattern warrants further investigation.

**Figure 12:**
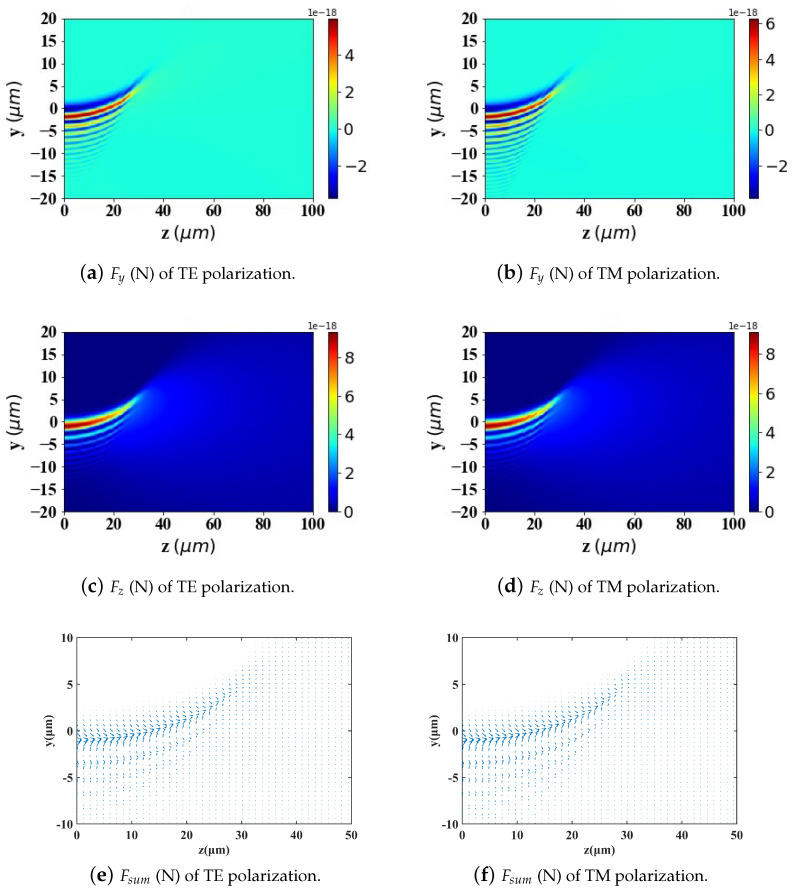
The same as in Figure 9 but set the refractive index of the environment to 1.45.

**Figure 13:**
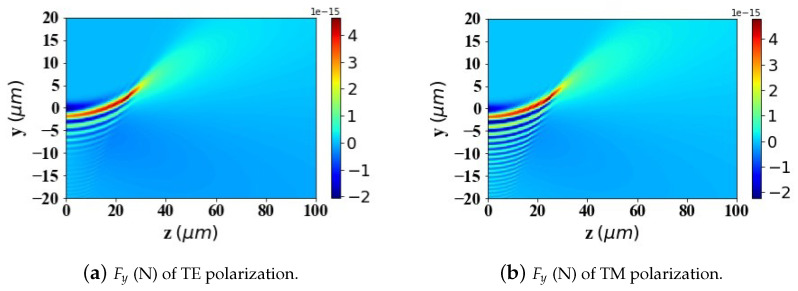

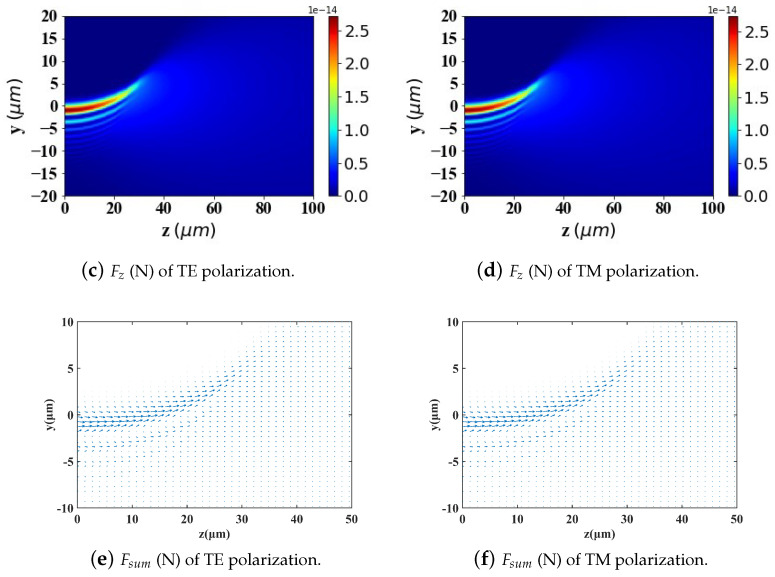
The same as in Figure 10 but set the refractive index of the environment to 1.45.

**Figure 14:**
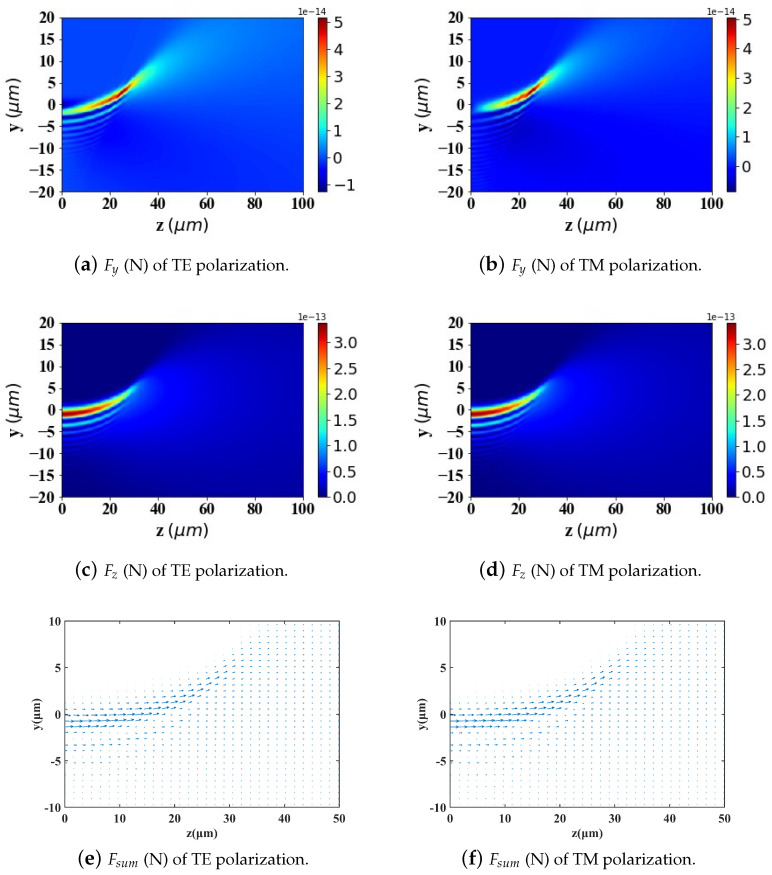
The same as in Figure 11 but set the refractive index of the environment to 1.45.

In the calculations presented in Figure 9, Figure 10 and Figure 11, the particle is assumed to be in the vacuum. To better reflect practical application scenarios, the refractive index of the surrounding medium is set to 1.45 (approximately the refractive index of whole blood) in Figure 12, Figure 13 and Figure 14, simulating the optical forces acting on magnetic nanoparticles when suspended in blood.

By comparing the results from Figure 9, Figure 10 and Figure 11 with those from Figure 12, Figure 13 and Figure 14, it can be concluded that the refractive index of the environment significantly influences the magnitude of the optical radiation force, which is closely related to the size of the particles. When the particle radius is 10 nm, which is much smaller than the wavelength of the incident Airy light sheet, the radiation force experienced by particles in an environment with a refractive index of 1.45 (including the transverse gradient force $F_{y}$ and the longitudinal scattering force $F_{z}$) is less than that experienced by particles in an environment with a refractive index of 1. Conversely, when the particle radius increases to 100 nm, approximately one-tenth of the wavelength, the longitudinal scattering force $F_{z}$ on particles in a refractive index of 1.45 is smaller; however, the transverse gradient force $F_{y}$ is greater than that experienced in an environment with a refractive index of 1. Furthermore, when the particle radius increases to 200 nm, the longitudinal scattering forces $F_{z}$ in both environments are roughly equivalent. In contrast, the transverse gradient force $F_{y}$ is larger in the environment with a refractive index of 1.45. Thus, it is evident that as the size of magnetic particles increases, the radiation force experienced by particles in an environment with a refractive index of 1.45 increases more significantly. This indicates that larger magnetic particles have a distinct advantage in an environment with a refractive index of 1.45, allowing them to experience stronger radiation forces.

To present the data more intuitively, panels (e) and (f) of Figure 9, Figure 10, Figure 11, Figure 12, Figure 13 and Figure 14 illustrate vector diagrams of the resultant forces $F_{y}$ + $F_{z}$ in the *y* and *z* directions. In these diagrams, the arrows indicate the direction of the resultant force acting on the particles, while the length of the arrows represents the magnitude of this force. Figure 9 shows that when the particle radius is 10 nm, the resultant force directs the particle toward the region of highest intensity in the Airy light-sheet, specifically its main lobe and some side lobes. Since the energy of the Airy light-sheet is primarily concentrated in the main lobe, this area exhibits the highest power, resulting in the maximum optical force. In Figure 10 and Figure 11, the longitudinal scattering force in the *z*-direction is significantly greater than the lateral gradient force in the *y*-direction; therefore, the overall direction of the resultant force aligns predominantly along the *z*-axis, causing the particle to be propelled in the direction of beam propagation.

### 3.2. Optical Torque

This section focuses on the phenomenon of optical spin torque (OST), which manifests exclusively in the case of a transverse magnetic (TM) polarized incident Airy light-sheet. Consequently, this section investigates the scenario where the structured illumination field is TM-polarized and proceeds to compute and analyze the OST acting on a magneto-dielectric spherical particle.

**Figure 15:**
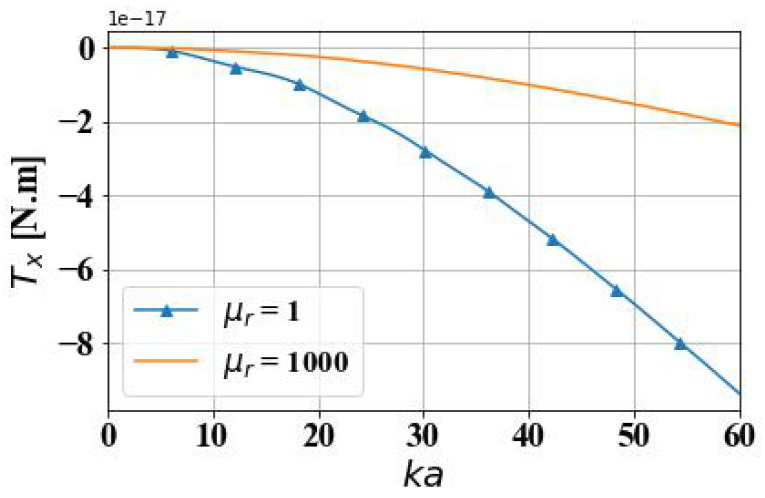
Comparison of OST on dielectric ($\mu_{r}$ = 1) and magnetic ($\mu_{r}$ = 1000) spheres.

Figure 15 presents a comparative analysis of the optical spin torque (OST) imparted by an Airy light-sheet on two distinct spherical particles: a dielectric particle with a relative permeability of $\mu_{r}=1$ and a magneto-dielectric particle characterized by a relative permeability of $\mu_{r}=1000$. It illustrates the variation in the torque component $T_{x}$ as the particle radius increases, with the torque measured in Newton-meters (N·m). The range of the optical size parameter ka for the particle is 0 to 60, corresponding to a maximum particle radius of approximately ten times the wavelength. The results show that OST is negative, indicating that the spherical particle can rotate clockwise under the action of the spin torque. As the particle radius increases, the magnitude of $T_{x}$ exhibits an increasing trend. In general, interactions between conventional light beams and particles primarily produce positive torque. Positive torque follows right-handed chirality, resulting in a counterclockwise rotation of particles. In contrast, negative torque introduces an opposite rotational direction, enabling clockwise particle rotation. In optical manipulation, positive and negative torques can achieve bidirectional control over micro- and nanoparticles or microstructures, thereby allowing precise dynamic control. This bidirectional control is precious in optical trapping and manipulation, where the interplay between positive and negative torques stabilizes the particle’s position or rotation direction, significantly enhancing manipulation precision. In medical applications, such as targeted drug delivery and intracellular nanomanipulation, this dual-directional control allows for highly accurate particle positioning and rotation, facilitating the precise targeting and controlled release of therapeutic agents at the cellular level.

Through numerical simulations, it is found that the OST acting on a magnetic spherical particle in an Airy light-sheet is smaller than the OST acting on a dielectric spherical particle. This implies that dielectric spherical particles have a more significant advantage in applications that require the exertion of rotational torque on particles. On the other hand, combined with the simulation results from the previous section, magnetic spherical particles experience larger radiation forces in the electromagnetic field distribution generated by an Airy light-sheet, compared to dielectric spherical particles, making them easier to confine and propel. However, the torque generated by the Airy light-sheet acting on magnetic spherical particles is smaller than those acting on dielectric spherical particles, making it more difficult to rotate magnetic spherical particles relative to dielectric spherical particles. Therefore, in practical applications, the choice of particle material should be based on specific requirements. If a larger rotational torque needs to be exerted on the particles, dielectric spherical particles may be a more suitable choice. Conversely, if the primary requirement is to exert a larger confinement or propulsion force on the particles, magnetic spherical particles may have an advantage. Through the reasonable design of the particle material and optical field parameters, precise control of the motion state of microparticles can be achieved.

**Figure 16:**
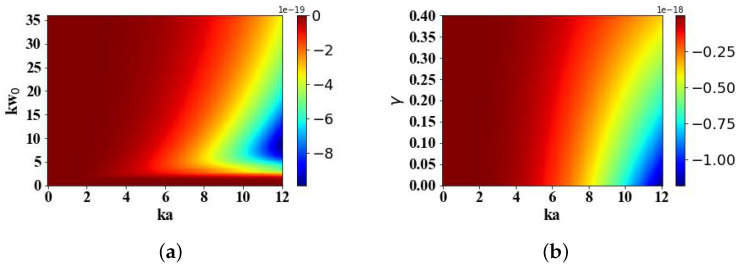
The influence of the incident Airy light-sheet parameters on $T_{x}$ (N·m). (**a**) The effects of $k\omega_{0}$ on $T_{x}$. (**b**) The effects of γ on $T_{x}$.

Figure 16 presents a comprehensive analysis of the optical spin torque (OST) acting on a magneto-dielectric spherical particle, investigating the influence of three key parameters: the optical size parameter ka of the particle, spanning a range from 0 to 12; the transverse scale parameter $k\omega_{0}$ of the Airy light-sheet, varying between 0 and 35; and the attenuation parameter γ of the Airy beam, ranging from 0 to 0.4. When the transverse scale $k\omega_{0}$ is less than 2, the torque component $T_{x}$ is zero. As $k\omega_{0}$ continues to increase, the magnitude of the torque $T_{x}$ first increases and then decreases, reaching its maximum value when $k\omega_{0}$ is approximately 0.75 times ka. The computational analysis reveals that an augmentation in the attenuation parameter γ is accompanied by a concomitant diminution in the intensity of the electromagnetic field. Consequently, this attenuation manifests as a corresponding reduction in the magnitude of the torque component $T_{x}$ experienced by the particle.

**Figure 17:**
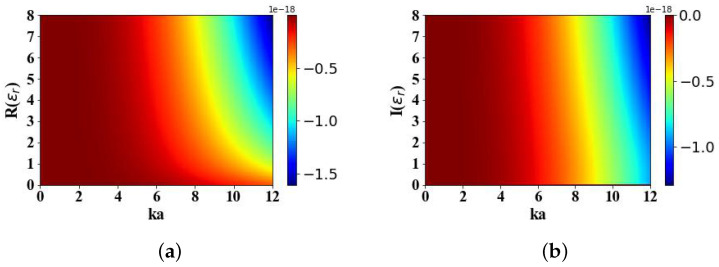
The influence of the relative permittivity of magnetic spherical particles on $T_{x}$ (N·m). (**a**) The effects of the real part of the relative permittivity on $T_{x}$. (**b**) The effects of the imaginary part of the relative permittivity on $T_{x}$.

Figure 17 calculates the impact of the real (ranging from 0 to 8) and imaginary (ranging from 0 to 8) parts of the magnetic particle’s permittivity on the OST experienced in the electromagnetic field distribution generated by an Airy light-sheet. This study reveals that an augmentation in both the real and imaginary components of the permittivity is accompanied by a concomitant escalation in the magnitude of the torque component $T_{x}$ experienced by the particle.

**Figure 18:**
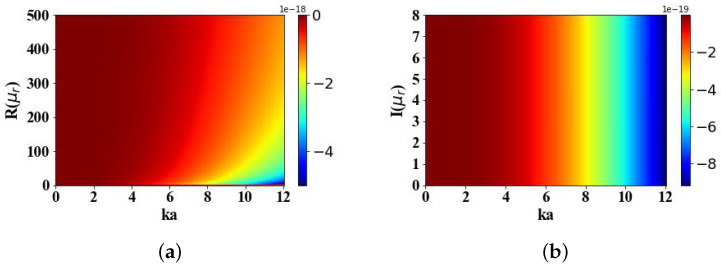
The influence of the relative permeability of magnetic spherical particles on $T_{x}$ (N·m). (**a**) The effects of the real part of the relative permeability on $T_{x}$. (**b**) The effects of the imaginary part of the relative permeability on $T_{x}$.

Figure 18 calculates the effects of the real (ranging from 0 to 500) and imaginary (ranging from 0 to 8) parts of the magnetic particle’s permeability on the OST experienced in the electromagnetic field distribution generated by an Airy light-sheet. An increase in the real part of the permeability results in a decrease in the magnitude of the torque $T_{x}$. However, variations in the imaginary part of the permeability have no impact on the magnitude of the torque.

**Figure 19:**
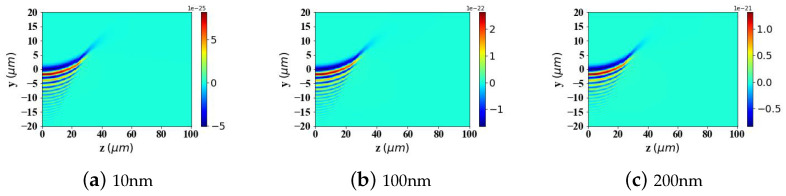
Two-dimensional spatial distribution of torque component $T_{x}$ (N.m) exerted by an Airy light-sheet on a magnetic spherical particle. (**a**) The magnetic spherical particle radius is 10 nm. (**b**) The magnetic spherical particle radius is 100 nm. (**c**) The magnetic spherical particle radius is 200 nm.

Figure 19 calculates the spatial distribution of the OST in the yz-plane for magnetic particles with sizes of 10 nm, 100 nm, and 200 nm, respectively. The results show that the distribution pattern of the torque component $T_{x}$ is similar to that of the incident Airy light-sheet. Similarly, larger particles experience greater radiation torques. The figure exhibits both positive and negative torque values, which can cause the particles to rotate in either the clockwise or counterclockwise direction.

**Figure 20:**
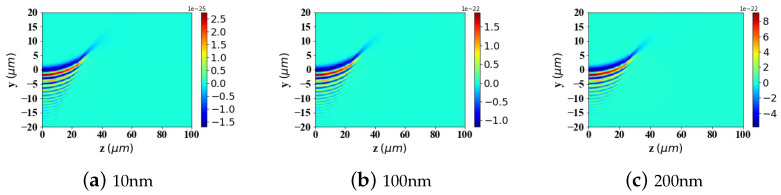
The same as in Figure 19 but set the refractive index of the environment to 1.45.

Figure 20 illustrates the optical spin torque experienced by particles in an environment with a refractive index of 1.45. Compared to the results shown in Figure 19, it can be observed that when particles are placed in an environment with a refractive index of 1.45, the optical spin torque they experience is less than that of particles in an environment with a refractive index of 1. Furthermore, this phenomenon is independent of the particle size.

## 4. Conclusions

This study systematically explores the interaction between Airy-light sheet and magnetic spherical particles, providing new theoretical perspectives and technical pathways for applying magnetic nanoparticles in targeted drug delivery systems. The following conclusions can be drawn: As the particle radius increases, both the optical radiation force and torque exhibit an increasing trend, with the resulting torque being negative, which causes the particles to rotate clockwise. The transverse scale parameter $k\omega_{0}$ of the Airy light-sheet have a minimal impact on the longitudinal scattering force $F_{z}$, primarily affecting the gradient force $F_{y}$. When the $k\omega_{0}$ of the Airy light-sheet increases, both the $F_{y}$ and torque $T_{x}$ initially increase before subsequently decreasing. Furthermore, an increase in the attenuation parameter γ leads to a reduction in the magnitudes of both the radiation force and torque $T_{x}$, with changes in the γ having a more significant effect on the $F_{y}$ than on the $F_{z}$. The polarization state of the incident field has little effect on the calculated results of the radiation force. As the real part of the permittivity increases, the $F_{y}$ decreases while the longitudinal scattering force $F_{z}$ increases, resulting in a larger magnitude for torque $T_{x}$. The imaginary part of the permittivity has almost no impact on the radiation force; however, an increase in this imaginary part will lead to an increase in the magnitude of torque $T_{x}$. Finally, when the real part of the magnetic permeability coefficient increases, the gradient force $F_{y}$ increases. In contrast, the longitudinal scattering force $F_{z}$ gradually decreases, reducing the magnitude of torque $T_{x}$. Notably, the imaginary part of the magnetic permeability coefficient does not significantly affect the optical forces and torques acting on magnetic particles.

We hope that this work will further advance the field, promote the widespread application of magnetic nanoparticles in biomedicine, and bring new breakthroughs in disease diagnosis and treatment. Additionally, this research offers significant theoretical references for manipulating other types of micro/nanoparticle systems using Airy light-sheet, potentially expanding the application scope of structured light fields in biomedicine and nanotechnology. However, it should be noted that while this study primarily focuses on the mechanical responses of magnetic spherical particles in Airy light fields, magnetic nanoparticles in practical applications often exhibit more complex shapes and structures. Therefore, future research needs to further explore the behavior of non-spherical magnetic nanoparticles in structured light fields and the impact of particle interactions on mechanical responses. Moreover, the complexity of the biological environment should also be considered in theoretical models to more accurately predict and optimize the movement and action of magnetic nanoparticles within the body.

## Data Availability

The original contributions presented in the study are included in the article, further inquiries can be directed to the corresponding author.

## Abbreviations

The following abbreviations are used in this manuscript:

MRI	Magnetic resonance imaging
OST	Optical spin torque
TE	Transverse electric
TM	Transverse magnetic
OF	Optical radiation force
OT	Optical radiation torque
BSCs	Beam shape coefficients

## References

[1] Mahmoudi M., Sant S., Wang B., Laurent S., Sen T. (2011). Superparamagnetic iron oxide nanoparticles (SPIONs): Development, surface modification and applications in chemotherapy. Adv. Drug Deliv. Rev..

[2] Shen L., Li B., Qiao Y. (2018). Fe_3_O_4_ Nanoparticles in Targeted Drug/Gene Delivery Systems. Materials.

[3] Tietze R., Zaloga J., Unterweger H., Lyer S., Friedrich R.P., Janko C., Pöttler M., Dürr S., Alexiou C. (2015). Magnetic nanoparticle-based drug delivery for cancer therapy. Biochem. Biophys. Res. Commun..

[4] Monaco I., Arena F., Biffi S., Locatelli E., Bortot B., La Cava F., Marini G.M., Severini G.M., Terreno E., Comes Franchini M. (2017). Synthesis of Lipophilic Core–Shell Fe_3_O_4_@SiO_2_@Au Nanoparticles and Polymeric Entrapment into Nanomicelles: A Novel Nanosystem for in Vivo Active Targeting and Magnetic Resonance–Photoacoustic Dual Imaging. Bioconjugate Chem..

[5] Ma M., Yan F., Yao M., Wei Z., Zhou D., Yao H., Zheng H., Chen H., Shi J. (2016). Template free synthesis of hollow/porous organosilica–Fe_3_O_4_ hybrid nanocapsules toward magnetic resonance imaging-guided high-intensity focused ultrasound therapy. ACS Appl. Mater. Interfaces.

[6] Nan X., Zhang X., Liu Y., Zhou M., Chen X., Zhang X. (2017). Dual-Targeted Multifunctional Nanoparticles for Magnetic Resonance Imaging Guided Cancer Diagnosis and Therapy. ACS Appl. Mater. Interfaces.

[7] Hajiaghajani A., Hashemi S., Abdolali A. (2017). Adaptable setups for magnetic drug targeting in human muscular arteries: Design and implementation. J. Magn. Magn. Mater..

[8] Al Faraj A., Shaik A.S., Shaik A.P., Al Sayed B. (2014). Enhanced magnetic delivery of superparamagnetic iron oxide nanoparticles to the lung monitored using noninvasive MR. J. Nanoparticle Res..

[9] Kayal S., Bandyopadhyay D., Mandal T.K., Ramanujan R.V. (2011). The flow of magnetic nanoparticles in magnetic drug targeting. RSC Adv..

[10] Lübbe A.S., Bergemann C., Riess H., Schriever F., Reichardt P., Possinger K., Matthias M., Dörken B., Herrmann F., Gürtler R. (1996). Clinical experiences with magnetic drug targeting: A phase I study with 4-epidoxorubicin in 14 patients with advanced solid tumors. Cancer Res..

[11] Depalo N., Iacobazzi R.M., Valente G., Arduino I., Villa S., Canepa F., Laquintana V., Fanizza E., Striccoli M., Cutrignelli A. (2017). Sorafenib delivery nanoplatform based on superparamagnetic iron oxide nanoparticles magnetically targets hepatocellular carcinoma. Nano Res..

[12] Liu Y.L., Chen D., Shang P., Yin D.C. (2019). A review of magnet systems for targeted drug delivery. J. Control. Release.

[13] Mishima F., Nakagawa K., Chuzawa M., Mori T., Akiyama Y., Nishijima S. (2012). Precise control of the drug kinetics by non-invasive magnetic drug delivery system. IEEE Trans. Appl. Supercond..

[14] Takeda S., Mishima F., Fujimoto S., Izumi Y., Nishijima S. (2007). Development of magnetically targeted drug delivery system using superconducting magnet. J. Magn. Magn. Mater..

[15] Liu J.F., Jang B., Issadore D., Tsourkas A. (2019). Use of magnetic fields and nanoparticles to trigger drug release and improve tumor targeting. Wiley Interdiscip. Rev. Nanomed. Nanobiotechnol..

[16] Spoială A., Ilie C.I., Motelica L., Ficai D., Semenescu A., Oprea O.C., Ficai A. (2023). Smart Magnetic Drug Delivery Systems for the Treatment of Cancer. Nanomaterials.

[17] Mody V.V., Cox A., Shah S., Singh A., Bevins W., Parihar H. (2014). Magnetic nanoparticle drug delivery systems for targeting tumor. Appl. Nanosci..

[18] Berry M.V., Balazs N.L. (1979). Nonspreading wave packets. Am. J. Phys..

[19] Broky J., Siviloglou G.A., Dogariu A., Christodoulides D.N. (2008). Self-healing properties of optical Airy beams. Opt. Express.

[20] Chu X., Wen W. (2014). Quantitative description of the self-healing ability of a beam. Opt. Express.

[21] Kotlyar V.V., Kovalev A.A. (2014). Airy beam with a hyperbolic trajectory. Opt. Commun..

[22] Forbes A., de Oliveira M., Dennis M.R. (2021). Structured light. Nat. Photonics.

[23] Otte E., Denz C. (2020). Optical trapping gets structure: Structured light for advanced optical manipulation. Appl. Phys. Rev..

[24] Siviloglou G.A., Christodoulides D.N. (2007). Accelerating Finite Energy Airy Beams. Opt. Lett..

[25] Siviloglou G.A., Broky J., Dogariu A., Christodoulides D.N. (2007). Observation of Accelerating Airy Beams. Phys. Rev. Lett..

[26] Baumgartl J., Mazilu M., Dholakia K. (2008). Optically mediated particle clearing using Airy wavepackets. Nat. Photonics.

[27] Kim K.Y., Kim S. (2016). Spinning of a submicron sphere by Airy beams. Opt. Lett..

[28] Lu W., Chen J., Lin Z., Liu S. (2011). Driving a dielectric cylindrical particle with a one dimensional Airy beam: A rigorous full wave solution. Prog. Electromagn. Res..

[29] Mitri F.G., Li R., Yang R., Guo L., Ding C. (2016). Optical pulling force on a magneto-dielectric Rayleigh sphere in Bessel tractor polarized beams. J. Quant. Spectrosc. Radiat. Transf..

[30] Mitri F.G. (2017). Optical pulling force and torques on Rayleigh semiconductor prolate and oblate spheroids in Bessel tractor beams. J. Quant. Spectrosc. Radiat. Transf..

[31] Li R., Yang R., Ding C., Mitri F.G. (2017). Optical torque on a magneto-dielectric Rayleigh absorptive sphere by a vector Bessel (vortex) beam. J. Quant. Spectrosc. Radiat. Transf..

[32] Barton J., Alexander D., Schaub S. (1989). Theoretical determination of net radiation force and torque for a spherical particle illuminated by a focused laser beam. J. Appl. Phys..

[33] Mitri F.G. (2021). Longitudinal and transverse PAFs for an absorptive magneto-dielectric circular cylinder in light-sheets of arbitrary wavefronts and polarization. Appl. Opt..

[34] Jiang Y., Chen H., Chen J., Ng J., Lin Z. (2015). Universal relationships between optical force/torque and orbital versus spin momentum/angular momentum of light. Physics.

[35] Jackson J.D. (2021). Classical Electrodynamics.

[36] van de Hulst H.C. (1981). Light Scattering by Small Particles.

[37] Bohren C.F., Huffman D.R. (2008). Absorption and Scattering of Light by Small Particles.

[38] Song N., Wei B., Li R., Sun H., Wei B., Zhang S., Zhang J., Mitri F.G. (2020). Optical torque on an absorptive dielectric sphere of arbitrary size illuminated by a linearly-polarized Airy light-sheet. J. Quant. Spectrosc. Radiat. Transf..

[39] Ye Q., Lin H. (2017). On deriving the Maxwell stress tensor method for calculating the optical force and torque on an object in harmonic electromagnetic fields. Eur. J. Phys..

